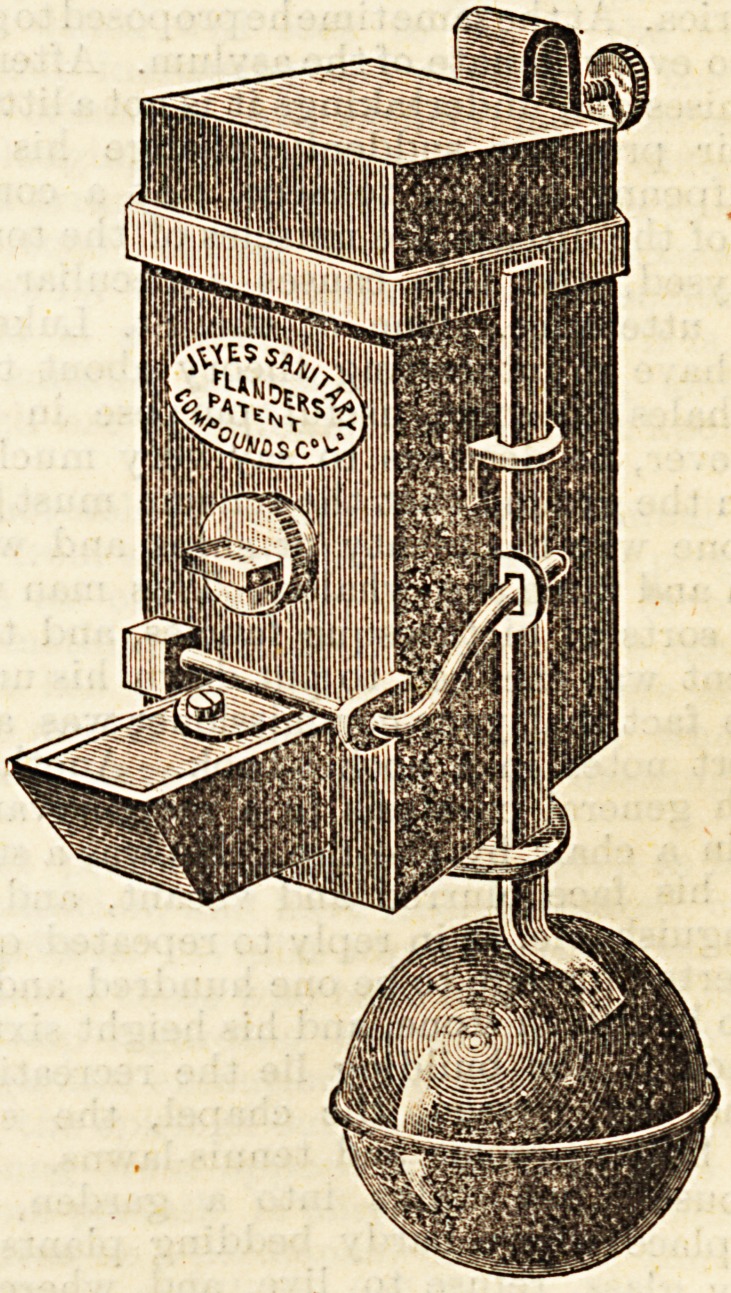# A Novelty in Disinfectants

**Published:** 1890-01-18

**Authors:** 


### A Novelty in Disinfectants.

Nobody doubts nowadays that necessity is the mother of invention. Prof. Flower believes that what he would call "pure scientists" have done more for the world than scientists who have worked for bread-and-butter or for fame. We do not agree with him. All kinds of practical inventions and improvements have been made in workshops, in factories, in engine-rooms, and at cottage firesides. A Watt, a Faraday, an Edison, an Arkwright, and a thousand others have each and all belonged to the class of bread-and-butter scientists. Disinfectants, which were luxuries thirty years ago, are universal necessities to-day. They are among the things that have won their way to fame by proved worth and efficiency. How much they have helped towards the practical banishment of typhoid fever, diphtheria, and many minor ills from large tracts of country it is impossible to say. What can be said with truth is that, thanks to disinfectants and other sanitary appliances, zymotic diseases are steadily on the decrease from year to year.

The great value of disinfectants being known, the next important step is to ensure their general distribution and use. No doubt all sensible people, being convinced of their value, take all necessary trouble to ensure their use. But what is the proportion of sensible people to the actual population? If Carlyle may be trusted, it is distressingly small; and even if he be held an exaggerator, it must still be admitted that the number of careless people, of lazy people, and of stupid people is very considerable indeed. Whilst he, therefore, who invents a good disinfectant, does well; he who invents an efficient method of distributing it without trouble to the user, does better still. That is exactly what Messrs. Jeyes, who make the "Perfect Purifiers," have accomplished. They have done well in making an excellent disinfectant, but they have done better in inventing, or having had invented for them, a little machine which will distribute any quantity of disinfectant through the drains, automatically and without trouble to anybody.

We annex a drawing of the automatic distributor. It is simplicity itself in construction, and may be worked by a child. In using the distributor, there are practically only two things to be done: first, to fill it carefully with the disinfectant, and then to attach it, by the hook provided for that purpose, to the cistern or other vessel in which the water requiring the purifier is placed. Once fixed, the machine works automatically, and every time the closet is used, or a tap turned for the discharge of water, exactly so much of the disinfectant as is necessary is liberated, and no more. The double purpose, therefore, of efficiency and economy is served in the most perfect way. The distributors are, of course, made of all sizes to suit every capacity of cistern, and being simple in construction they are quite inexpensive and not at all likely to get out of order. We have on previous occasions expressed our satisfaction with the disinfectants manufactured by Messrs. Jeyes. In bringing their distributor into the market they have conferred a still greater obligation on all town dwellers, as well as on occupiers of large country houses. We hardly need advise all those who believe in and use disinfectants to ensure their scientific and efficient distribution by fixing an automatic distributor of suitable size in every house and stable cistern they may possess.

## Figures and Tables

**Figure f1:**
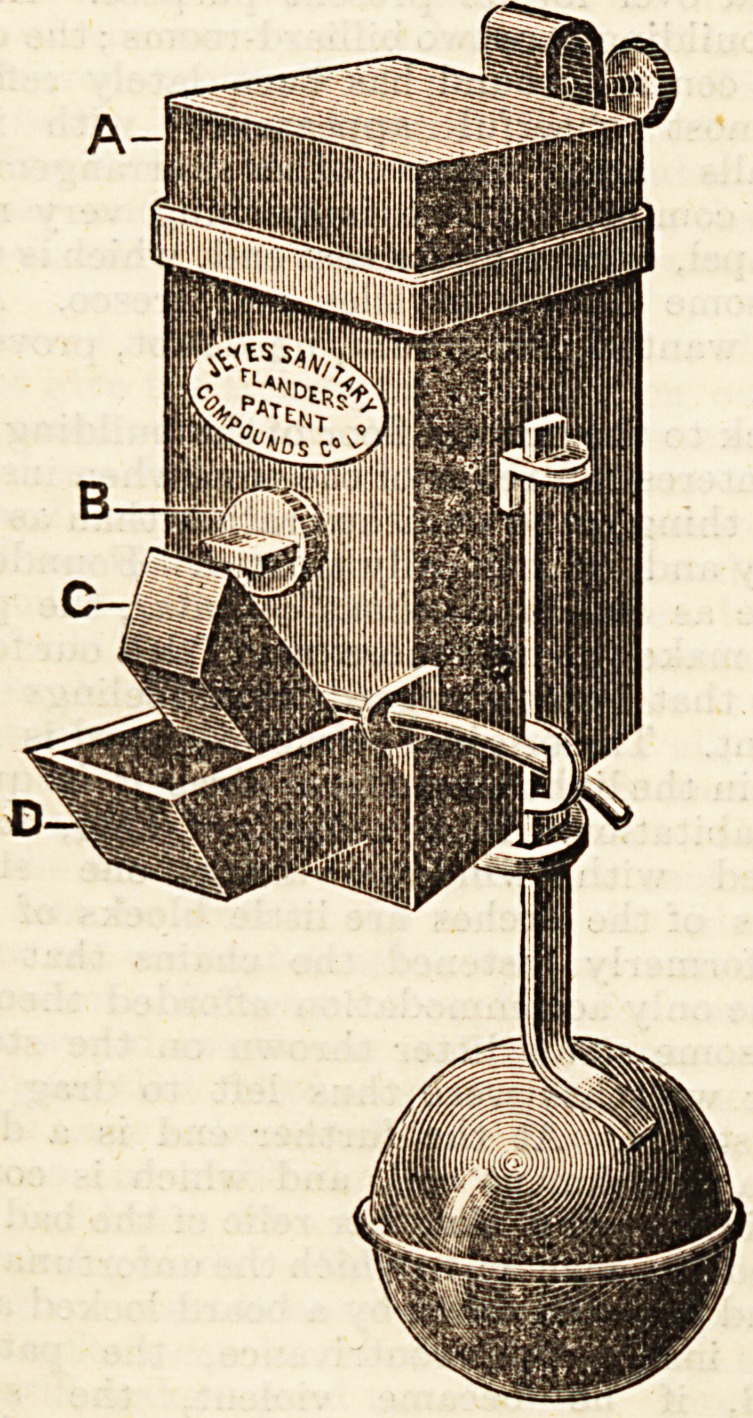


**Figure f2:**